# Effects of high-fat diet and physical activity on pyruvate dehydrogenase kinase-4 in mouse skeletal muscle

**DOI:** 10.1186/1743-7075-9-53

**Published:** 2012-06-09

**Authors:** Rita Rinnankoski-Tuikka, Mika Silvennoinen, Sira Torvinen, Juha J Hulmi, Maarit Lehti, Riikka Kivelä, Hilkka Reunanen, Heikki Kainulainen

**Affiliations:** 1 Neuromuscular Research Center, Department of Biology of Physical Activity, University of Jyväskylä, Jyväskylä, Finland; 2 Department of Biological and Environmental Sciences, University of Jyväskylä, Jyväskylä, Finland; 3 LIKES Research Center for Sport and Health Sciences, University of Jyväskylä, Jyväskylä, Finland

**Keywords:** Skeletal muscle, Mitochondria, Lipids, Glucose, Fuel switching

## Abstract

**Background:**

The expression of PDK4 is elevated by diabetes, fasting and other conditions associated with the switch from the utilization of glucose to fatty acids as an energy source. It is previously shown that peroxisome proliferator-activated receptor γ coactivator 1α (PGC-1α), a master regulator of energy metabolism, coactivates in cell lines pyruvate dehydrogenase kinase-4 (PDK4) gene expression via the estrogen-related receptor α (ERRα). We investigated the effects of long-term high-fat diet and physical activity on the expression of PDK4, PGC-1α and ERRα and the amount and function of mitochondria in skeletal muscle.

**Methods:**

Insulin resistance was induced by a high-fat (HF) diet for 19 weeks in C57BL/6 J mice, which were either sedentary or with access to running wheels. The skeletal muscle expression levels of PDK4, PGC-1α and ERRα were measured and the quality and quantity of mitochondrial function was assessed.

**Results:**

The HF mice were more insulin-resistant than the low-fat (LF) -fed mice. Upregulation of PDK4 and ERRα mRNA and protein levels were seen after the HF diet, and when combined with running even more profound effects on the mRNA expression levels were observed. Chronic HF feeding and voluntary running did not have significant effects on PGC-1α mRNA or protein levels. No remarkable difference was found in the amount or function of mitochondria.

**Conclusions:**

Our results support the view that insulin resistance is not mediated by the decreased qualitative or quantitative properties of mitochondria. Instead, the role of PDK4 should be contemplated as a possible contributor to high-fat diet-induced insulin resistance.

## Background

A multitude of studies have postulated that obesity and the metabolic syndrome caused by sedentary lifestyle and western diet decrease the capacity of skeletal muscles to oxidize the accumulated lipids [1,2]. Previously this has been proposed to occur by decreased mitochondrial content as well as mitochondrial biogenesis and function [[3-8] suggesting an association between mitochondrial dysfunction and insulin resistance, the qualitative and quantitative changes in mitochondria being potentially the ultimate cause [9,10]. However, recent studies have convincingly shown that high-fat diet actually increases mitochondrial biogenesis and fatty acid oxidative capacity in skeletal muscle [11-13] and that lipid-induced insulin resistance in the absence of physical activity is strongly associated to incomplete β-oxidation and mitochondrial overload or “mitochondrial stress” [14]. Mitochondrial defects per se, e.g. deficient electron transport chain, do not seem to be the cause of insulin resistance [15].

Although reduced muscle mitochondrial content and function have been proposed to be a consequence of physical inactivity and sedentary lifestyle, exercise efficiently stimulates muscle oxidative capacity and thus corrects the imbalance between fatty acid uptake and oxidation [16-18]. Furthermore, physical activity reduces the reliance on carbohydrates, thus increasing the proportion of fatty acids used as an energy source and enhancing muscle fatty acid oxidation, especially during submaximal exercise [19,20].Peroxisome proliferator-activated receptor γ coactivator-1α (PGC-1α) is a potential main regulator of the metabolic program that has been shown to be acutely activated by exercise training and down-regulated by high-fat feeding and sedentary lifestyle [21]. PGC-1α has known roles in mitochondrial biogenesis and fatty acid oxidation. The ability of PGC-1α to co-activate the orphan nuclear receptor ERRα (estrogen- related receptor) results in the activation of a broad mitochondrial program, including the induction of oxidative phosphorylation and mitochondrial biogenesis [22-25]. It has been demonstrated both in humans [26] and in rodents [27,28] that the expression of PGC-1α is induced by exercise [29,30] after the activation of PGC-1α promoter [28]. Despite the many functions of PGC-1α in overall energy homeostasis, its function as a potential regulator in glucose utilization pathways is not well characterized [31].

The pyruvate dehydrogenase kinases (PDKs) regulate the activity of pyruvate dehydrogenase complex (PDC), which catalyzes the oxidative decarboxylation of pyruvate in the glucose oxidation process. The isoform PDK4 is highly expressed in liver, heart and skeletal muscle and is regulated by exercise. Its expression is elevated with diabetes, fasting and other conditions associated with the switch from the utilization of glucose to fatty acids as an energy source [32,33]. It has been suggested that insulin resistance is associated with dysregulation of the PDC in skeletal muscle and that excess insulin would on the other hand down-regulate the expression of PDK4 [25,34,35]. Interestingly, transcription factor ERRα and transcriptional co- activator PGC-1α both induce PDK4 gene expression independently [31,36]. In addition, it has been shown that PGC-1α is recruited to the PDK4 promoter by ERRα, which stimulates further the expression of PDK4 [31,37,38]. Our primary aim was to study the effects of high-fat diet and physical activity on the expression of PDK4 and aspects of its regulation. We hypothesized that when dietary carbohydrates are replaced by fatty acids as a fuel for oxidation in muscle, the expression of PDK4 is increased, and this elevation is regulated by the PGC-1α/ERRα-pathway. Secondly, we studied the effect of high-fat diet and physical activity on the amount and function of mitochondria in skeletal muscle.

## Methods

### Animals and diets

Male C57BL/6 J mice (n = 58) were obtained from Taconic (Ejby, Denmark) at the age of 6 weeks and were individually housed in standard conditions (temperature 22°C, humidity 50 ± 10%, light from 8:00 am to 8:00 pm). After one week of adaptation to new environment, the mice were matched for body-weight (20.8 ± 1.4 g) and divided into four groups. The mice received for 19 weeks *ad libitum* either a lard- based purified high-fat diet (61% of energy from fat, 19% protein, 20% carbohydrates 5.16 kcal/g; D12492-Euro) to induce obesity and insulin resistance, or a low-fat diet as a control diet (10% of energy from fat, 19% protein, 71% carbohydrates, 3.78kcal/g; D12450-Euro, Purina Mills TestDiet®, PMI® Nutrition International,Richmond, IN, USA)*.* The nutritional profile of the fat content of the two diets was as follows (high-fat diet/low-fat diet): cholesterol 229/18 ppm, linoleic acid 3.97/1.39%, linolenic acid 0.36/0.19%, arachidonic acid 0.05/0.00%, omega-3 fatty acid 0.36/0.19%, total saturated fatty acids 10.54/1.14%, total monounsaturated fatty acids 10.84/1.30%. The groups of low-fat fed (LF) or high-fat fed (HF) mice were either sedentary (LFsed or HFsed) or physically active (LFexe or HFexe) throughout the experiment. Mice were housed individually in cages and the physically active mice had access to a running wheel, as previously described [39]. The amount of running was monitored via a computerized system across the study period. All mice were weighed and food consumption was monitored at three-week intervals. Feeding efficiency was calculated (weight gained in mg per kilocalories consumed), but no numerical results are presented and only significant differences are mentioned in the results. The protocols were approved by the Animal Care and Use Committee of the University of Jyväskylä.

**Figure 1:**
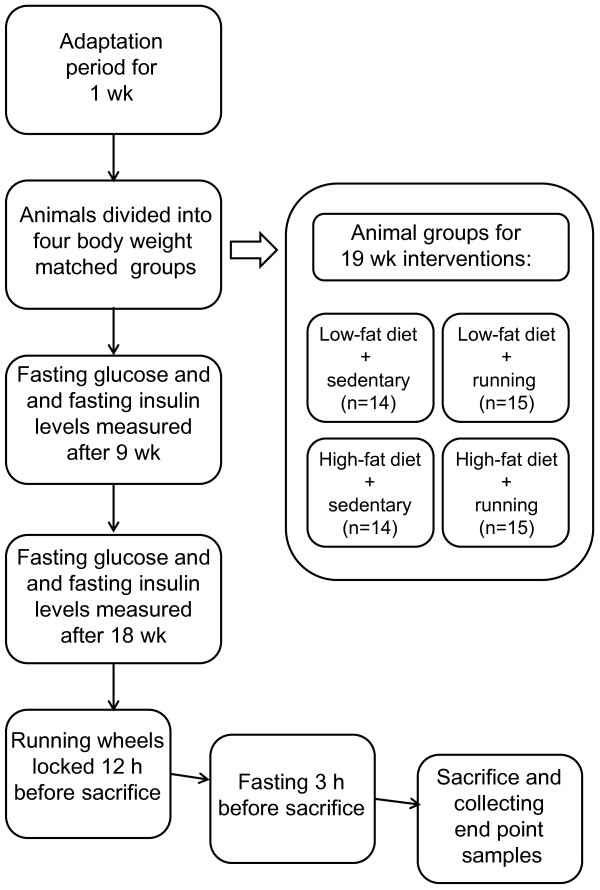
**Summary of study design.** Graph summarizing the experiment set up and data collection points.

The running wheels were locked for 12 hours before sacrifice. After 3-hours’ fasting the animals were weighed and then sacrificed by cervical dislocation. Blood and serum samples were collected for the triglyceride, cholesterol and free fatty acid measurements. The muscles extensor digitorum longus (EDL), soleus, gastrocnemius and quadriceps femoris (QF) and epididymal fat pads were excised from the animals, weighed and prepared for further analysis. Total RNA isolation was done from the left gastrocnemius. The muscle oxygen consumption measurements were done from the right QF and homogenates for the Western blotting and histological samples were prepared from the left QF. Histological samples were transversally oriented and mounted on OCT compound (Tissue Tek, Sakura Finetek Europe) and snap frozen in isopentane cooled with liquid nitrogen (−160°C). Electron microscopic analyses were done from the soleus muscle. The experiment set up and data collection points are summarized in Figure 1.

### Serum analyses

After overnight fasting, a blood sample was collected at intervention weeks 9 and 18 and the blood glucose level was determined (HemoCue, Ängelholm, Sweden). Insulin was analyzed with an Ultra Sensitive Rat Insulin ELISA Kit according to manufacturer’s protocol (Crystal Chem Inc., Downers Grove, IL, USA). Insulin resistance was estimated by multiplying the fasting values of glucose and insulin. Triglycerides, total cholesterol and free fatty acids were measured from the end-point serum samples, of which triglycerides and cholesterol were measured using the VITROS DT60 II Chemistry System (Ortho-Clinical Diagnostics, Rochester, NY, USA). The Wako NEFA C test kit (Wako Chemicals GmbH, Neuss, Germany) scaled down to a microplate format was used to determine free fatty acids (FFA).

### RNA extraction and cDNA synthesis

Total RNA was isolated from (approximately 50 mg of) the gastrocnemius with Trizol reagent (Invitrogen, Carlsbad, CA, USA) according to manufacturer’s instructions. Muscle samples were homogenized with a FastPrep (Bio101 Systems, USA) tissue homogenizer by using Lysing Matrix D (Q-Biogene, USA). The concentration and purity of RNA were determined photometrically at wavelengths of 260 nm and 280 nm. The integrity of RNA was checked with agarose gel electrophoresis. Five micrograms of total RNA was reverse transcribed to synthesize cDNA (SuperScript III Reverse Transcriptase kit, Invitrogen). For efficient mRNA transcription, a mixture of oligo primers (Oligomer, Helsinki, Finland), consisting of 20 dT residues followed by two additional nucleotides, which anneal only at the 5’ end of the poly(A) tail of mRNA, was used.

### Real-time quantitative PCR

The mRNA expression levels of ERRα, PCG-1α and PDK4 were determined with the ABI 7300 Real-Time PCR system (Applied Biosystems, Foster City, CA, USA). The TaqMan primer and probe sets were designed and synthesized by Applied Biosystems. The gene bank accession numbers and Applied Biosystems assay IDs, respectively were: NM_007953.2 and Mm00433142_m1 (ERRα), NM_008904.1 and Mm01208833_m1 (PGC-1α), NM_013743.2 and Mm00443326_m1 (PDK4). The PCR cycle parameters used were: +50°C for 2 min, +95°C for 10 min, 40 cycles at +95°C for 15 s, and +60°C for 1 min. All samples were analyzed in triplicate. The gene expressions were normalized using a Quant-iT™ PicoGreen® assay (Invitrogen) according to manufacturer’s instructions. The PicoGreen method was used to quantitate the total amount of RNA-cDNA-hybrids from the solution of reverse- transcribed mRNA products [40].

### Western blotting

The QF muscle samples were hand-homogenized in 4% homogenization buffer [10% SDS (w/v), 40 mM DTT, 5 mM EDTA, 0.1 M Tris–HCl pH 8 and protease inhibitors 40 μg/ml aprotinin, 80 μg/ml PMSF and 40 μg/ml leupeptin (Sigma, Saint Louis, USA)]. Western immunoblot analyses from the muscle lysates (samples containing 20 μg of total protein) were done as previously described [41,42]. Briefly, PVDF membranes were incubated overnight at 4°C with rabbit primary antibodies against PGC-1α (1:1000, Calbiochem, Merck KGaA, Darmstadt, Germany), PDK4 and ERRα (1:1200 and 1:3000 respectively, Novus Biologicals, Littleton, CO, USA), and with goat antibody against cytochrome c (CytC, 1:2000 Santa Cruz Biotechnology Inc., Santa Cruz, CA, USA). Membranes were incubated with horseradish peroxidase- conjugated secondary anti-rabbit or anti-goat IgG antibody (Jackson ImmunoResearch Laboratories, PA, USA) diluted 1:50 000 or 1:70 000, respectively, in TBS-Tween (0.1%) with 2.5% milk for 1 h followed by washing in TBS-T. Preliminary experiments confirmed a proportional linear relationship between protein loaded and, especially, Ponceau S but also α-actin (1:20 000, Sigma) in quantification between 5 and 60 μg, demonstrating the suitability of Ponceau S to be used as a method to control for loading [42]. Proteins were visualized by ECL according to manufacturer's protocol (SuperSignal West femto maximum sensitivity substrate, Pierce Biotechnology, Rockford, IL, USA) and quantified using ChemiDoc XRS Quantity One software (version 4.6.3. Bio-Rad, UK).

### Image analysis of SDH activity

Serial cross-sections (8 μm) from the QF muscle were cut in a cryomicrotome (−25°C). The activity of succinate dehydrogenase (SDH) was used as a marker for muscle fiber oxidative capacity as described by Pette and Tyler [43]*.*

The SDH-stained cross-sections (n = 4-12 animals/group) were captured in full color using light microscopy (Olympus BX-50, Olympus Optical, Tokyo, Japan). Digitally captured images (magnification 20 x) with a minimum of three fields-of-view per muscle cross-section were processed and analyzed using ImageJ software (NIH, Bethesda, MD, USA). The images were converted to 8-bit gray-scale (range of grey levels 0–255) images. An intensity threshold representing minimal intensity values corresponding to SDH activity was set manually and uniformly used for all images (least oxidative gray levels 46–90; most oxidative 140–255). The three intensity scaled fractions representing different oxidative capacities of fibers were expressed as the percentage of the measured area.

### Electron microscopic analysis of mitochondrial content

Pieces of soleus (n = 5 animals/group) were fixed with 3% glutaraldehyde in 0.1 M phosphate buffer for 2–2.5 h at +4°C, and post-fixed with 1% osmium tetroxide in the same buffer at +4°C for 1 h. The specimens were stained in uranyl acetate, dehydrated in ethanol and embedded in LX-112 (Ladd). Semithin sections were cut, stained with toluidine blue and examined with light microscope to optimize the transverse orientation. Thereafter, ultrathin sections were cut, mounted on grids and stained with uranyl acetate and lead citrate. Micrographs were taken from the best section of each block with a Jeol JEM-1200 electron microscope at 2500 x primary magnification. It was checked that micrographs were taken from different cells (10–13 cells/section) and that sarcolemmal areas were included. In total 343 micrographs were analyzed using AnalySIS software (Olympus). The amount of subsarcolemmal mitochondria was expressed as mitochondrial area (μm^2^) and related to the length of sarcolemma (μm).

### Measurements of mitochondrial respiration

The homogenization of QF muscle samples and isolation for the mitochondrial respiration measurements was done mainly according to Wardlaw *et al.*[44] with minor modifications. Briefly, mitochondrial respiration rates (30 μl of freshly prepared mitochondria) were measured at 25°C with a Clark-type oxygen electrode (Hansatech Instruments Ltd, England) in a reaction medium. Respiration rates were recorded in the presence of complex I substrates pyruvate (5 mM) and malate (2.5 mM). State 3 respiration was initiated by adding 150 mM ADP (1.5 mM in buffer). Oxygen consumption was related to the protein content of the suspension determined in triplicates according to manufacturer’s instructions (BCA assay kit, Pierce). Mitochondrial respiration rates in the QF muscle homogenates were measured using the same procedure as the respiration of isolated mitochondria.

### Statistical analysis

All data are presented as mean ± SD. A repeated general linear model was used with weight gain, feeding efficiency and weekly running distance as parameters. Two-way ANOVA was used to determine the effect of diet (with 2 levels: low-fat diet and high- fat diet), exercise (with 2 levels: with and without voluntary running), and their interaction with the measured variables, as previously described [39]. Differences between the means of the intervention groups were evaluated, and the significance of differences was determined by Bonferroni post hoc testing. All statistical analyses were carried out using PASW statistics software release 18.0 (IBM Corporation, Armonk, NY, USA). Differences of *P <* 0.05 were considered significant.

## Results

### Food consumption, body mass and tissue weight

**Figure 2:**
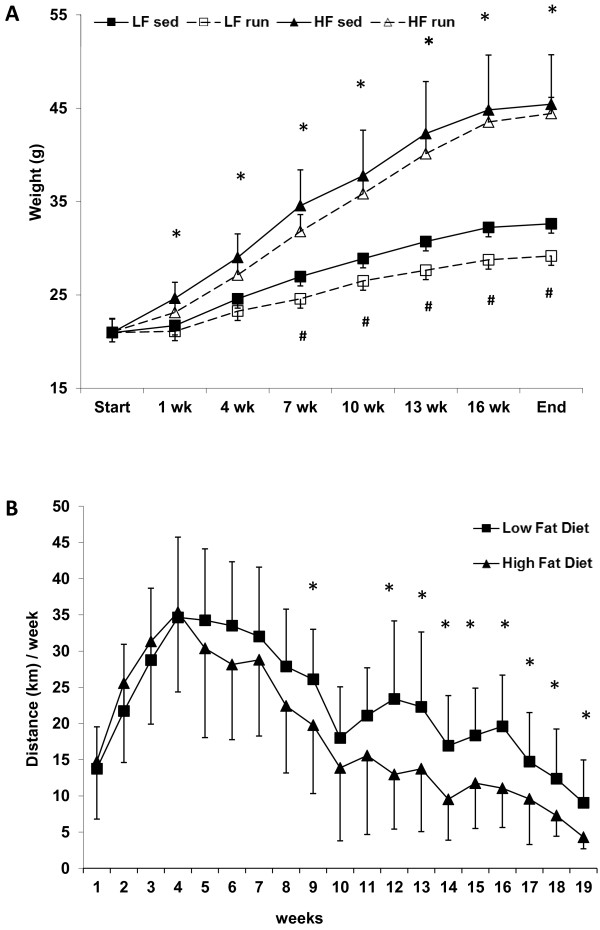
**Body weight gain and voluntary running of the mice during the 19-week experiment. (A)** After only 1 week, the body weight of low fat (LF) and high fat (HF) mice differed significantly (asterisk). After 7 weeks, the LFsed mice were significantly heavier than the LFexe mice (#). n = 14-15 animals/group. * *P* < 0.001, # *P* < 0.05. **(B)** Voluntary running distances per week on the running wheel during the 19-week intervention. After 9 weeks and after 12 weeks until the end of the experiment, the running distances differed significantly between the two diet groups. n = 14-15 animals/group. * P < 0.05 was considered significant. Results are means (±SD).

The development of body weight is shown in Figure 2A. After only 1 week of intervention, significantly higher body weight was observed in the HF-fed mice compared to LF-fed mice. Thereafter, the body weight of the HF mice increased continuously during the experiment. After seven weeks of intervention a significant difference in body weight between the sedentary and their respective running groups was seen throughout the rest of the intervention. Consistent with their body weight, the HF mice had heavier epididymal fat pads and quadriceps femoris muscles (QF) than the LF mice (Table 1).

**Table 1:** Physiological characteristics

**Basic data**	**Low-fat diet**		**High-fat diet**		**ANOVA*P*-value**		
	**Sedentary (n=14)**	**Running (n=15)**	**Sedentary (n=14)**	**Running (n=15)**	Diet	Running	Diet*Running
Weight (g)^#^	32.6 ± 2.86	29.2 ± 1.72^**^	45.4 ± 5.29^***^	44.4 ± 3.12^***,¤¤¤^	<0.001	0.008	0.051
Fat (mg)	799.13 ± 345.1	424.04 ± 65.4^*^	1767.38 ± 383.2^***^	1925.06 ± 541.0^***,¤¤¤^	<0.001	0.275	0.009
Gastrocnemius (mg)	144.98 ± 12.6	141.12 ± 7.7	151.34 ± 8.6	154.81 ± 7.4^**,¤¤¤^	<0.001	0.936	0.137
Quadriceps femoris (mg)	206.53 ± 10.1	211.35 ± 11.8	220.61 ± 13.0^**^	228.23 ± 11.0^***,¤¤¤^	<0.001	0.045	0.646
EDL (mg)	12.96 ± 1.4	12.47 ± 1.1	13.19 ± 1.7	12.83 ± 1.1	0.721	0.500	0.404
Soleus (mg)	10.85 ± 1.6	11.81 ± 1.3	11.14 ± 1.2	13.80 ± 1.6^***,§§§,¤¤¤^	0.004	<0.001	0.028

^#^Logarithmic transformation for normality and comparison.

Body weight, epididymal fat mass, and the masses of gastrocnemius, quadriceps femoris, EDL and soleus muscles were measured at the end of the 19-week experiment. The muscle masses are average of both limbs. * = vs. LFsed (*P* < 0.05), ** = vs. LFsed (*P* < 0.01), *** = vs. LFsed (*P* < 0.001), §§§ = vs. HFsed (*P*<0.001), ¤¤¤ = vs. LFexe (*P* < 0.001). Results are means (±SD).

Feeding efficiency varied in the different groups throughout the experiment. The feeding efficiency values of the HF mice ranged from 16.75 ± 4.55 mg/kcal to 7.55 ± 4.15 mg/kcal during the three-week monitoring intervals, and were significantly higher than those of the LF mice (9.78 ± 2.25 mg/kcal and 3.85 ± 3.18 mg/kcal, respectively). Running induced a slight decrease in feeding efficiency in the LF mice.

### Voluntary running

After four weeks of running, both the LF and HF groups reached their maximum weekly running distance, which then decreased gradually (Figure 2B). Consistent differences in the weekly running distance were observed after 12 weeks, the running distance of LF mice being significantly higher than that of HF mice. However, no statistically significant difference between the groups in total cumulative running distance (LF 422 ± 108 km, HF 339 ± 136 km) was observed.

### Blood glucose, insulin and lipid profile

**Figure 3:**
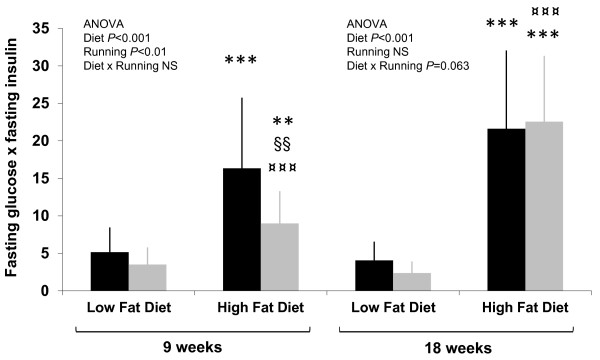
**Estimated insulin resistance after 9 and 18 weeks of intervention.** The insulin resistance of high fat diet groups differed significantly from the low fat diet groups. Additionally, after 18 weeks on HF diet, running no longer had an ameliorating effect. n = 14–15 animals/group. ** = vs. LFsed (*P* < 0.01), *** = vs. LFsed (*P* < 0.001), ¤¤¤ = vs. LFexe (*P* < 0.001), §§ = vs. HFsed (*P* < 0.01), NS =non-significant (*P* > 0.1). *Black bars* = sedentary, *grey bars* = running.

The fasting glucose levels were significantly higher in the HF compared to LF mice. There was also a difference within the group of HF mice, with the runners having higher fasting glucose (Table 2). The HF animals had significantly higher fasting insulin levels compared to LF animals. Estimated insulin resistance indicated that already after 9 weeks on the HF diet the HF mice were more insulin resistant than the LF mice and that a significant positive effect of running was seen in both diet groups (Figure 3). After 18 weeks on the HF diet the HF mice were significantly more insulin resistant than the LF mice. However, no statistical difference between the sedentary and running animals in the HF diet group was observed thereafter, which is concomitant with the decreased running activity seen in Figure 2B.

**Table 2:** Blood profiles of the mice after the 19-week experiment

**Basic data**	**Low-fat diet**		**High-fat diet**		**ANOVA*P*-value**		
	**Sedentary (n=14)**	**Running (n=15)**	**Sedentary (n=14)**	**Running (n=15)**	**Diet**	**Running**	Diet*Running
Total cholesterol (mmol/l)^#^	2.99 ± 0.88	2.70 ± 0.36	4.90 ± 0.54^***^	4.52 ± 0.49^***,¤¤¤^	<0.001	0.033	0.795
Triglycerides (mmol/l)	0.97 ± 0.23	1.05 ± 0.21	1.00 ± 0.24	0.90 ± 0.13^¤^	0.274	0.807	0.089
Free fatty acids (mmol/l)	0.82 ± 0.15	0.92 ± 0.16	0.49 ± 0.13^***^	0.44 ± 0.12^***,¤¤¤^	<0.001	0.572	0.108
Fasting glucose (mmol/l)	8.92 ± 1.17	8.45 ± 0.90	9.39 ± 1.12	10.53 ± 0.72^***,§§,¤¤¤^	<0.001	0.201	0.003
Fasting insulin (ng/ml)^#^	0.43 ± 0.24	0.27 ± 0.16^*^	2.25 ± 1.11^***^	2.14 ± 0.82^***,¤¤¤^	<0.001	0.125	0.112

^#^Logarithmic transformation for normality and comparison.

Fasting blood glucose and insulin were measured after 18 weeks. * = vs. LFsed(*P* < 0.05), *** = vs. LFsed (*P* < 0.001), §§ = vs. HFsed (*P* < 0.01), ¤ = vs. LFexe (*P* < 0.05), ¤¤¤ = LFexe (*P* < 0.001). Results are means (±SD).

The high-fat diet had an effect on total cholesterol and on free fatty acids (FFA), the cholesterol levels being higher and, somewhat unexpectedly, the FFA levels lower in the HF groups (Table 2). The HFexe and LFexe groups also differed in total cholesterol, FFA and triglyceride levels.

### mRNA expression

**Figure 4:**
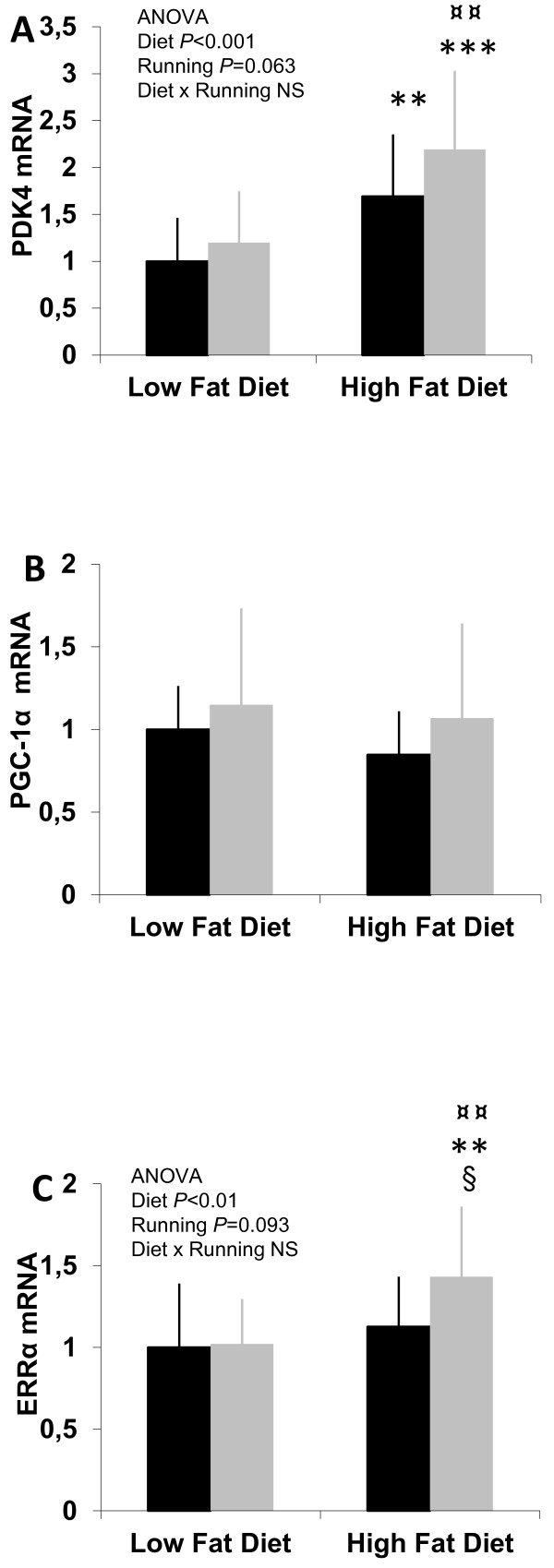
**The mRNA expression levels measured with quantitative RT-PCR in gastocnemius muscle.** (A) In the mRNA expression levels of PDK4 there was a statistical effect of diet. The expression levels were significantly higher in HF mice groups compared to LFsed animals and additionally in HFexe group compared to LFexe. PGC-1α (B) expression level differences did not reach statistical significance between any of the groups. Also the mRNA expression levels of ERRα (C) showed a statistical effect of diet. HFexe mice had significantly higher expression in ERRα compared to other groups. The results are expressed in relation to the LFsed mean value. n = 14-15 animals/group. ** = vs. LFsed (*P* < 0.01), *** = vs. LFsed (*P* < 0.001), § = vs. HFsed (*P* < 0.05), ¤¤ = vs. LFexe (*P* < 0.01), NS = non-significant (*P* > 0.1). *Black bars* = sedentary, *grey bars* = running.

The expression level of PDK4 (Figure 4A) in the HF-fed animals, especially when combined with running, was significantly higher than in the LFsed animals. No change in the expression of PGC-1α mRNA levels after HF diet or chronic exercise was observed (Figure 4B). The expression of ERRα (Figure 4C) was significantly up-regulated after HF feeding combined with running than it was in the three other groups (P <0.05-0.01).

### Protein expression

**Figure 5:**
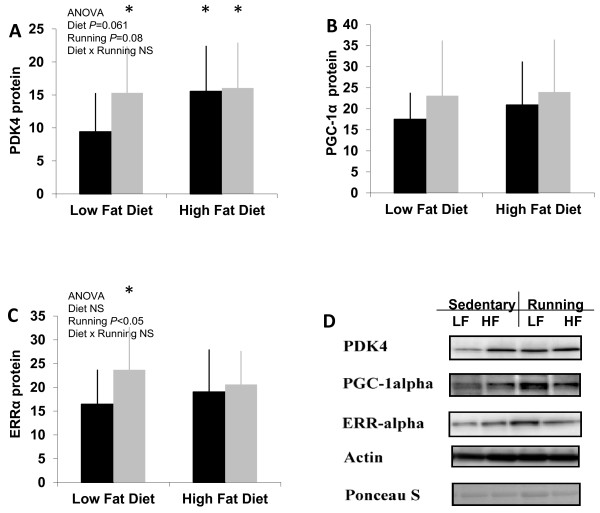
**The protein expression levels in QF muscle.** PDK4 **(A)** expression showed higher expression levels in all other groups, when compared to LFsed mice. PGC-1α **(B)** showed no statistical differences between the groups. ERRα **(C)** expression was considered to show a statistical effect of running. The protein expression results are normalized to the mean value of LFsed. **(D)** Representative Western blot images. n = 14-15 animals/group. * = vs. LFsed (*P* <0.05), NS = non-significant (*P* > 0.1). *Black bars* = sedentary, *grey bars* = running.

Exercise and diet both significantly increased the expression of PDK4 (P < 0.05) compared to LFsed mice, but exercise had no additional effect on the HF mice (Figure 5A).

Although the change was most pronounced in PDK4, also PGC-1α (Figure 5B) and ERRα (Figure 5C) proteins showed a similar trend: both running and high-fat feeding increased the expression of each protein, but high-fat feeding combined with running had no additive effect on the protein expressions (no difference between the HF groups). PGC-1α expression showed a slight, although not statistically significant, effect for diet and for running. Exercise increased the expression of ERRα in the LF mice (P < 0.05).

### Skeletal muscle oxidative capacity

**Figure 6:**
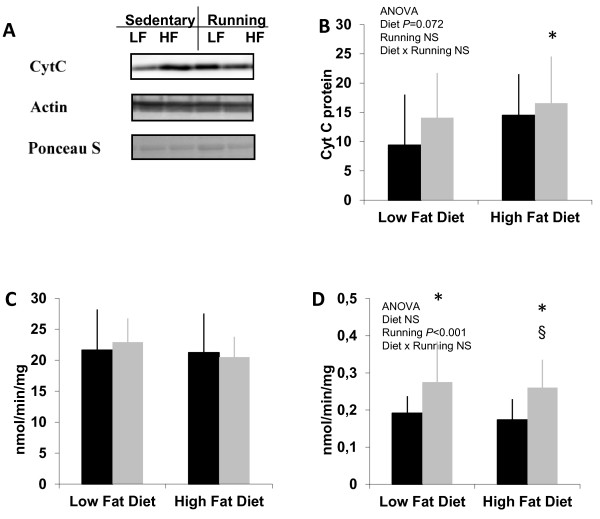
**Measures of estimated oxidative capacity in skeletal muscle.** Cytochrome *c* (CytC) content in Western blotting **(A)** when normalized to Ponceau S **(B)** showed a trend of HF diet main effect (*P* = 0.072). n = 14–15 animals/group. The oxygen consumption of mitochondria **(C)** in QF muscle did not show any statistical difference between the groups. n = 10–12 animals/group. **(D)** Oxygen consumption in muscle homogenates was higher in the running groups than in the respective sedentary groups. n = 10–12 animals/group. * = vs. LFsed (*P* < 0.05), § = vs. HFsed (*P* < 0.05), NS = non-significant (*P* > 0.1). *Black bars* = sedentary, *grey bars* = running.

Cytochrome c content measured by Western blotting showed no statistically significant differences between the groups (Figure 6A and 6B), although high-fat diet had nearly significant (P = 0.072) main effect and combination of HF and exercise showed significance compared to LF sedentary group. Oxygen consumption of the isolated mitochondria (Figure 6C) did not differ between the study groups. However, mitochondrial oxygen consumption in muscle homogenate (Figure 6D) was significantly increased in running groups (P < 0.02).

**Figure 7:**
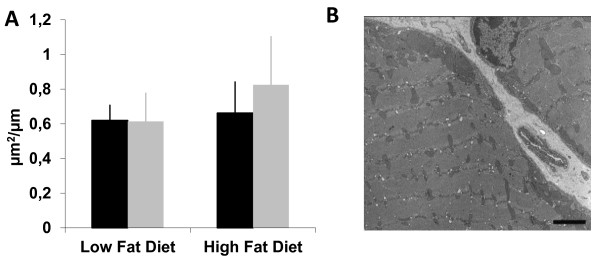
**Oxidative capacity estimated by electron microscopy. (A)** There were no statistical differences between any of the groups in the area of subsarcolemmal mitochondria relative to the length of the sarcolemma when electron microscopic micrographs were analysed. n = 5 animals/group. *Black bars* = sedentary, *grey bars* = running. **(B)** Electron microscopic image from the subsarcolemmal mitochondria. Scale bar 2 μm.

The soleus muscle was analyzed by electron microscopy, which showed clusters of mitochondria beneath the sarcolemma, often located near the capillaries and lipid droplets (Figure 7A and B). The area occupied by mitochondria was ~20% larger in the HFexe mice than HFsed mice and ~25% larger than in the LF mice (Figure 7A), although the differences were not statistically significant. The ultrastructure of mitochondria was normal in all groups.

Results from the SDH-staining of QF muscles (Table 3) showed that the HFexe mice had a larger proportion of the most oxidative fiber type area than the HFsed mice (P <0.05).

**Table 3:** Oxidative capacity estimated by SDH staining

SDH stain intensity	**Low-fat diet**		**High-fat diet**	
	**Sedentary (n=4)**	**Running (n=12)**	**Sedentary (n=8)**	**Running (n=7)**
Least oxidative (%)	32.5 ± 18.0	39.4 ± 15.2	49.8 ± 18.4	30.3 ± 26.2
Intermediate (%)	42.8 ± 12.3	35.8 ± 7.3	33.4 ± 11.8	39.0 ± 15.6
Most oxidative (%)	24.7 ± 8.7	24.8 ± 11.1	16.8 ± 7.8	30.7 ± 16.8^§^

According to the SDH staining of the QF muscle, HFexe mice yielded the highest measure of SDH activity,i.e. the largest area of the most oxidative fiber types (vs.HFsed P < 0.05). § = vs. HFsed (*P* < 0.05).

## Discussion

In the present study we observed concomitantly with explicit insulin resistance an up- regulated PDK4 expression along with less prominent ERRα expression in response to the high-fat diet and/or to voluntary exercise. We also found that high-fat diet did not alter the oxidative capacity of isolated mitochondria or oxygen consumption in the muscle homogenate. Voluntary running exercise improved insulin sensitivity during the first 9 weeks of the high-fat diet, but no longer after 18 weeks, concomitantly with decreased running activity. The effects of exercise on the mitochondrial parameters were comparable or greater to those of the high-fat diet, but in most cases exercise and high-fat diet did not have additional/synergistic effects.

In addition to its ability to exert effects on oxidative metabolism in muscle [45], it has been suggested that PGC-1α controls skeletal muscle glucose metabolism by increasing the amount of PDK4 via a PGC-1α/ERRα-dependent mechanism [31]. This is further supported by the finding that ERRα recruits PGC-1α to the PDK4 promoter [37,38]. Our results show distinct effects of high-fat diet and voluntary running on PDK4 protein expression and, more elaborately, an additive effect of both HF diet and voluntary running on mRNA expression but not on protein expression. Although we did not measure the activity of PDC in our experiment, it is known that PDK4 negatively regulates the PDC, thus inhibiting the entry of pyruvate to the Krebs cycle [46]. In addition, PDK4 has been found to be a contributor to lipid-induced changes of glucose metabolism in rodent and human studies [47-49]. We speculate that increased PDK4 expression after high-fat feeding and exercise is due to previous increase in PGC-1α and ERRα expression, which subsequently blunts cellular glucose oxidation. Our results suggest that in addition to molecular and cellular level *in vitro*[31], PGC- 1α/ERRα-dependent regulation of PDK4 expression also may operate *in vivo* in skeletal muscle.

Previous studies have demonstrated that a high-fat diet can increase the biogenesis of mitochondria and fatty acid oxidative capacity in skeletal muscle [12,13]. It can be suggested that not only in the state of increased energy demand, such as exercise, but also in the case of constantly increased energy supply with high fatty acid availability, the oxidation of fatty acids can be intensified. This paradigm is supported by the present data, which shows improved total mitochondrial capacity in response to a high-fat diet for 19 weeks. These results contradict previous findings according to which a high-fat diet decreases the capacity of muscles to oxidize the accumulated lipids [1,2], which would occur owing to the decreased number of mitochondria, as reported in the offspring of type 2 diabetic parents [6].

PGC-1α is considered the master regulator that coordinates the gene expression of oxidative metabolism as well as mitochondrial biogenesis in skeletal muscle [50,51]. In our study the effect of chronic high-fat feeding for 19 weeks had no effect on the expression of PGC-1α. This is in contrast to a previous study that showed decreased PGC-1α expression in muscle after 1 week on a HF diet that persisted down-regulated over 11 weeks [52]. In other studies a high-fat diet for 4–5 weeks has even increased muscle PGC-1α protein expression and the number of mitochondria [11,12]. These discrepancies may be partly due to differences in the fatty acid compositions of the diets, since it has been shown that, depending on their chain length and saturation level, fatty acids have greatly varying effects on PGC-1α expression [52]. PGC-1α mRNA and protein expression peak rapidly after a stimulus, such as an exercise bout [14,26,29] or an increase in the concentration of serum fatty acids [53]. After a period of adaptation, no change or only slight changes in PGC-1α mRNA and protein levels have been observed, after 4 weeks of high-fat diet [11] or, as in the present study, after a high-fat diet and/or exercise for 19 weeks. In addition to PGC-1α, ERRα, acting downstream of PGC-1α, is also a critical transcriptional regulator of mitochondrial biogenesis and cellular energy metabolism [24,28,31]. Moreover, ERRα is expressed in tissues demonstrating a high capacity for fatty acid β-oxidation [54,55]. In this study, we found a significant increase in ERRα mRNA expression after a high-fat diet combined with voluntary running, and in protein expression after a low-fat diet combined with running. We believe that the modest changes observed in PGC-1α and ERRα expression are remnants of previous high increases caused by every single exercise bout and/or dietary fatty acids. A limitation to our study is that we measured only the transcript levels of PGC-1α, but not alternative regulatory mechanisms. PGC-1α activity is also regulated by protein modifications, including phosphorylation, acetylation and ubiqitination [56].

High-fat feeding declines general physical activity in rodents [57,58]. Similarly, in this study high-fat feeding induced consistent reduction of wheel-running after 12 weeks of diet, although at the end of the experiment cumulative running distances did not statistically differ between LF and HF mice. Access to running wheels increases general cage activity and affect several components of energy balance (reviewed in Novak et al. [59]) that may have effects to the regulation of muscle metabolism. However, it is not possible to dissect the effects of these factors in this study. In this study the HF mice with or without exercise were severely insulin resistant, as indicated by their increased levels of fasting insulin and glucose, suggesting that they had developed a metabolic condition resembling metabolic syndrome or type 2 diabetes [60]. In our experiment, we found the plasma free fatty acid concentration to be significantly lower in the HF animals compared to LF animals. Conceivably, skeletal muscle had adapted to the chronic high-fat diet to be able to better extract and oxidize circulating lipids. The preference for fatty acids as an energy source is reflected in elevated blood glucose. Our data may suggest that both in chronic high-fat diet and in long-term exercise training, the switch of fuel usage from glucose to fatty acids is mediated by the elevated expression of PDK4. It is known that during long-term exercise or short-term fasting, the activity of PDC is attenuated in conjunction with increased fatty acid usage [61]. Accordingly, the expression of PDK4 is increased in fasting, diabetes and other conditions associated with switching from glucose oxidation to fatty acid oxidation [62].

What is the mechanism behind high-fat diet-induced insulin resistance? It has been shown that chronic high-fat diet-induced insulin resistance, unlike insulin resistance induced by acute increase in plasma free fatty acids (i.e. Randle glucose fatty acid cycle), is not rapidly reversible [63]. On the basis of our studies, we agree that most probably it is not the decrease in the amount or intrinsic function of mitochondria that leads to increased intramyocellular lipids [12,13]. Our data on insulin resistance and normal mitochondrial function support the idea that lipids themselves or metabolites of lipid metabolism attribute to impaired response to insulin, e.g. via altered cell membrane properties [64] or by affecting IRS phosphorylation and GLUT4 translocation [65]. Our data further suggest that the inhibition of pyruvate dehydrogenase by PDK4 is a possible contributor to insulin resistance. In this scenario high-fat diet-induced insulin resistance may be a consequence of the continuing regulatory process of PGC-1α/ERRα activated by chronic high fatty acid availability. Our data also show that voluntary running exercise improved insulin resistance only transiently during the 19-week high-fat diet, implying that the regulatory power of fatty acids is superior to exercise. On the other hand, the inability of exercise to improve insulin sensitivity after the 19 weeks of wheel running in the experiment might be due to the reduced amount of running during the latter half of the experiment. The role of fatty acids in insulin resistance is a complex process, with some fatty acids inducing and others reversing skeletal muscle insulin resistance [66], suggesting that a balanced fatty acid composition in the diet would be beneficial for optimal muscle cell metabolism and function.

## Conclusions

We conclude that a chronic high-fat diet does not have a negative effect on muscle mitochondrial function in spite of severe insulin resistance. This finding suggests that, contrary to frequent allegation, insulin resistance is not mediated by the decreased qualitative or quantitative properties of mitochondria. Instead, our data suggest that the role of PDK4 should be contemplated as a possible contributor to high-fat diet- induced insulin resistance.

## Competing interests

The authors declare that they have no competing interests.

## Authors’ contributions

RR-T participated in the design and execution of the study and drafted the manuscript, MS participated in the design and execution of the study, ST and HR participated in electron microscopic analyses, JJH participated in protein analysis, ML and RK participated in the execution of the study, HK participated in the design and coordination of the study and helped to draft the manuscript. All authors read, revised and approved the manuscript.
